# China’s process-related greenhouse gas emission dataset 1990–2020

**DOI:** 10.1038/s41597-023-01957-y

**Published:** 2023-01-25

**Authors:** Xiang Yu, Chang Tan

**Affiliations:** 1 University of Chinese Academy of Social Sciences, Beijing, 102488 China; 2 Research Institute for Eco-civilization (RIEco), Chinese Academy of Social Sciences, Beijing, 100710 China; 3 Department of Earth System Science, Ministry of Education Key Laboratory for Earth System Modeling, Institute for Global Change Studies, Tsinghua University, Beijing, 100084 China

**Keywords:** Climate-change mitigation, Climate-change mitigation

## Abstract

China’s industrial process-related Greenhouse Gas (GHG) emissions are growing rapidly and are already equivalent to 13–19% of energy-related emissions in the past three decades. Previous studies mainly focused on emissions from fossil fuel combustion, however, there are a broad range of misconceptions regarding the trend and source of process-related emissions. To effectively implement emission reduction policies, it is necessary to compile an accurate accounting of process-related GHG emissions. However, the incompleteness in scope, unsuitable emission factor, and delay in updates in the current emission inventory have led to inaccurate emission estimates and inefficient mitigation actions. Following the methodology provided by Intergovernmental Panel on Climate Change (IPCC), we constructed a time series inventory of process-related GHG emissions for 15 industrial products from 1990–2020 in China. This emission inventory covers more than 90% of China’s process-related GHG emissions. In our study, emission factors were adjusted to refer to the industrial production process, technology, and raw material structure in China, which has led to increased accuracy of emission accounting. The dataset can help identify the sources of process-related GHG emissions in China and provide a data base for further policy implications.

## Background & Summary

During the rapid industrialization and urbanization of the past decades, China’s industrial value-added accounted for 39% of the total national GDP and 26% of the global industrial value-added in 2020^1^. As one of the most carbon-intensive sectors in the world, the industry sector in China is the major emitter of both energy-related and process-related Greenhouse Gas (GHG) emissions. Previous studies mainly focused on fossil fuel combustion^2–5^, however, the emissions from industrial processes are generally misunderstood and overlooked, which may result in insufficient mitigation measures. The latest official emission data is from the statistics of *The People’s Republic of China’s Second Biennial Update Report on Climate Change*. It shows that China’s total industrial process-related GHG emissions were 1330 Mt in 2014, which accounts for a sizeable 14% of energy-related emissions^6^.

The Chinese central government aims to have CO_2_ emissions peak before 2030 and achieve carbon neutrality before 2060.To effectively implement emission reduction policies, it is necessary to compile an accurate record of process-related GHG emissions, however, there are limited studies about the industrial process-related emission accounting in China, and most of them only focus on cement production. For example, Ke *et al*.’s research concluded that the process-related CO_2_ emission from China’s cement industry ranged from 498 to 679 Mt in 2007^7^. Shen *et al*. concluded that more than 50% of total emissions from the primary cement production process are process-related emission in 2012^8^. According to Andrew’s study, China’s cement production accounted for 782 Mt of process CO_2_ emissions in 2018, accounting for 52% of global process-related emissions from cement production, due to China producing 56% of the world’s cement^9^. Liao *et al*. also estimate process-related CO_2_ emissions from China’s cement industry at the provincial level and the result shows that Guangdong and Anhui are the two provinces with highest cement process-related emission in 2019^10^.

The Cement is a major source of process-related emissions, but emissions from other industrial products also should be taken into consideration. Shan *et al*. focus on lime production and suggest the process-related emission from China’s lime industry was about 142 Mt in 2012^11^. Liu *et al*. measured the process-related carbon emissions of 5 industrial products including glass, soda ash, ammonia, calcium carbide and alumina from 1990 to 2013^12^. Based on Liu’s study, the research of Cui *et al*., provided an process-related emission inventory for 10 industrial products during 2003–2018^13^, and the total process-related emission for 10 industrial products in Cui’s research only accounts for 32% of the total process-related emission published in the *People’s Republic of China’s Second Biennial Update Report on Climate Change*^6^.

In summary, there are three main deficiencies in the present studies that focus on industrial process-related emissions in China: (1) The most studies focus on cement, alhough a few studies involve other products, the variety of product types is limited, resulting in an erroneous minimization of emissions from many industrial products. (2) The use of default emission factors, which do not account for China-specific production processes and technologies, leads to inconsistency between the emission results and true values. (3) The lack of continuous, up-to-date, and detailed carbon inventory lead to the key industrial process-related emissions being ignored when emission reduction policies are developed. For these reasons, there is an urgent need to quantify the GHG emissions from industrial processes in China to achieve the targets of carbon peaking and carbon neutrality.

In this study, we developed a process-related GHG emission inventory dataset of 15 industrial products in China from 1990 to 2020, and provided the most up-to-date and accurate emission data by prioritizing emission factors according to the energy and technology structure in China. The dataset in this study consists of not only emission inventory but also activity data and emission factors^14^.

## Methodology and Data Source

### Methodology

**Fig. 1:**
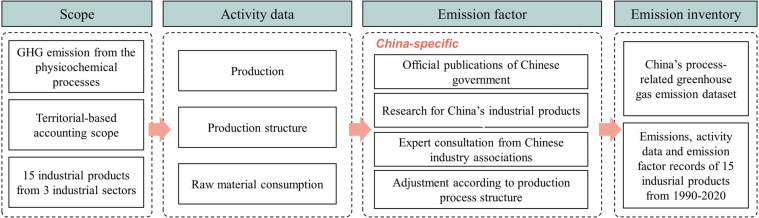
The framework of industrial process-related GHG emission dataset constructed.

In this study, we have calculated the process-related GHG emission for 15 industrial products in China from 1990–2020. Figure 1 demonstrates the industrial process and method of the emission accounting. To caculate the process-related GHG emission, we use the the activity data to multiply emission factor, and the scopes of accounting also need to be defined.

Industrial process-related GHG emissions are the emissions from the physicochemical processes which raw materials are transformed into products^15^. The fossil fuels burned for heat or power caused energy-related emission, which is excluded in process-related emissions. We adopted the territorial-based emission accounting boundary, defined as emissions that occur within the territorial and offshore areas under the jurisdiction of a nation^16^.

We select 15 industrial products in the mineral, chemical and metal industries. The emissions from these 15 industrial products accounts for more than 90% of the total industrial process-related emissions in China in 2014 which stated in *The People’s Republic of China Second Biennial Update Report on Climate Change*^6^. The 15 industrial products and their respective sectors are shown in Table 1.

**Table 1:** Industrial sector and products.

IPCC code	Industrial sector	Industrial products
2.A	Mineral industry	Clinker, Lime, Glass
2.B	Chemical industry	Methanol, Ammonia, Nitric acid, Adipic acid, Calcium carbide, Soda ash
2.C	Metal industry	Crude steel, Ferroalloys, Aluminum, Magnesium, Lead, Zinc

There are three methods to estimate emissions: (1) by emission factor, (2) by mass balance, (3) by measurement. The mass balance approach requires plant-specific material flow analysis of each production line which requires a massive quantity of data^15^. The measurement approach requires installing monitor equipment at each emission source^17^, and is therefore more costly. Compared to the other methods, the emission factor approach is the most widely and commonly applied emission accounting method since the data sources are usually publicly available, and its accuracy can be guaranteed if suitable emission factors are adopted. The *2006 IPCC Guidelines for National Greenhouse Gas Inventories* presents two methods for emission factor^15^: Tier 1 and Tier 2, which both estimate emissions based on production output and emission factors, however, Tier 2 uses emission factors from the country-specific investigation while Tier 1 use default global average value.

Considering the data availability and accounting accuracy, Tier 2 is used to estimate the process-related GHG emissions (Eq. (1)).

1 $$G{E}_{i}=A{D}_{i}\times E{F}_{i}$$

Where:

*GE*_*i*_ represents the GHG emissions from product *i* (in CO_2_ emission equivalent).

*AD*_*i*_ represents the activity data. The industrial product is represented by *i*.

*EF*_*i*_ represents the emission factor, which describes the GHG emissions from per unit of production of product *i*. The emission factors represented in CO_2_ emission equivalent  and  in their original gas type are both provided in the data file.

### Data source

In this study, activity data is the output of industrial products. We collected the output data from several statistical yearbooks of China, including *China Industry Statistical Yearbook*, *China Steel Industry Yearbook*, and *China Nonferrous Metal Industry Yearbook*, etc. The clinker production data is from the public report of the China Cement Association^18–21^. The lime and adipic acid production output estimates are collected from online information offered by United States Geological Survey^22^ and the website of Industry Information(www.chyxx.com).

Emission factors play a vital role in calculating the total emissions and are determined by the production process. The production processes of industrial products in China significantly differ from those in other countries. For example, the clinker to cement ratio is much lower in China compared to the global average level^9^. The chemical industry in China uses a large amount of coal as feedstocks^23^ while European countries depend more on natural gas^15^. China is also one of the few countries that adopts the electrolytic process for lead refinery^24^. The different production process would lead to different emission factors. Therefore, national-specific emission factors for China should be used for more accurate accounting.

The following two methods are used to correct the emission factors: (1) If an emission factor of the industrial product in China is available, we used this emission factor for the accounting. (2) If integrated emission factors in China are not available, then we calculate them according to the production process. Specifically, the weighted average of the emission factor of each production process is based on the prevalence of the production process for a specific industrial product in terms of percentage.

The Method (1) is firstly adopted for emission factors in a portion of the products. The emission factors from the GHG accounting guidelines published by Chinese government are in line with the actual situation in China. Therefore, priority was given to using these emission factors published by the National Development and Reform Commission (NDRC) of China in the *GHG Emission Accounting Guideline for 10 Industry-Specific Corporate* in 2020^25^ and then the *Guidelines for Provincial Greenhouse Gas Inventories* published in 2011^26^. Among the 15 products, emission factors for ferroalloy, perfluorochemicals (PFCs) emissions and anode effect of aluminum are from *GHG Emission Accounting Guideline for 10 Industry-Specific Corporate*. The emission factors of lime, 5 production routes of nitric acid, adipic acid, calcium carbide, limestone solvent use and the carbon content of pig iron and steel are from *Guidelines for Provincial Greenhouse Gas Inventories*. Whereas the emission factor for clinker production is obtained from Liu *et al*.’s study which uses the cement kiln dust correction factor to adjust it in China^27^. The emission factor for glass production is from Hu *et al*.’s study which applied a material inventory based on the field investigation of Chinese glass factories^28^.

The Method (2) is adopted to adjust emission factors for the remainder of the products. Suppose a product is produced by different processes, such as methanol, nitric acid, soda ash, lead and zinc, the emission factor of these products largely depend on the share of different processes. Therefore, we firstly collected the emission factors for different processes and calculated the weighted average of each production process to synthesize a new integrated emission factor. The emission factors of 5 production routes of nitric acid are collected from guidelines published by NDRC as stated in the above paragraph.

The emission factors of chemical products in China are distinct from those of other countries due to the different production routes and raw materials^23^, however, the emission factors of chemical products in China are not available from public data sources. Therefore, we investigated the emission factors of methanol and ammonia by consulting experts from the China Petroleum and Chemical Industry Federation (CPCIF), the emission factors of 5 key industrial processes for methanol production were identified, including methanol by natural gas (one-stage conversion), natural gas (two-stage conversion), bituminous coal, anthracite coal, and coke oven gas. The integrated emission factor of ammonia is also obtained from the consultation with CPCIF experts following the same procedure.

Among the three production processes of soda ash, only the natural soda process produces process-related emissions. The emission factor of the natural soda ash process is from *2006 IPCC Guidelines for National Greenhouse Gas Inventories*^15^. Then we obtained the percentage of natural soda ash in China from China Soda Industry Association (CSIA) and calculated the integrated emission factors of soda ash in China.

For the process-related emission factors of lead and zinc, Sjardin gives a complete review^29^ which is also cited and adopted by the IPCC’s emission accounting guidelines^15^. We obtained the original emission factor of different production routes of lead and zinc production from Sjardin’s study. The share of different processes in China’s lead and zinc production is sourced from the International Lead and Zinc Study Group^24^. The emission factors are weighted according to their percentages.

The emission factor of magnesium includes CO_2_ emission from electrolysis of magnesium chloride solution as well as the SF6 emissions which functions as a protective gas to prevent oxidation of magnesium. The CO_2_ emission factor of magnesium soured from Sjardin’s study^29^ and the SF6 emission factors are from *Guidelines for Provincial Greenhouse Gas Inventories*^26^. The adopted emission factors and data sources are shown in Table 2.

**Table 2:** Process-related GHG emission factors.

Industrial products	Process detail	Value	Unit	Source
**Mineral industry**				
Clinker	/	0.50	tCO_2_/t	^27^
Lime	/	0.68	tCO_2_/t	^26^
Glass	/	0.07	tCO_2_/t	^28^
**Chemical industry**				
Methanol	By natural gas	0.41	tCO_2_e/t	Expert consultation
	By bituminous coal	1.91	tCO_2_e/t	Expert consultation
	By anthracite	2.25	tCO_2_e/t	Expert consultation
	By coke oven gas	0.34	tCO_2_e/t	Expert consultation
Ammonia		2.97	tCO_2_/t	Expert consultation
Nitric acid	High pressure	0.53	tCO_2_e/t	^26^
	Medium pressure	3.12	tCO_2_e/t	^26^
	Normal pressure	2.58	tCO_2_e/t	^26^
	Double pressurized	2.12	tCO_2_e/t	^26^
	Comprehensive method	1.99	tCO_2_e/t	^26^
Adipic acid	/	77.65	tCO_2_e/t	^26^
Calcium carbide	/	1.15	tCO_2_/t	^26^
Soda ash	Natural soda	0.14	tCO_2_/t	^15^
	Solvay soda	0.00	tCO_2_/t	^15^
	Hou’s soda	0.00	tCO_2_/t	^15^
**Metal industry**				
Limestone use	/	0.43	tCO_2_/t Limestone	^26^
Carbon content of pig iron	/	4.10	%	^26^
Carbon content of steel	/	0.25	%	^26^
Ferroalloy	/	0.28	tCO_2_/t	^25^
Aluminum	Anode effect - CF4	0.23	tCO_2_e/t	^25^
	Anode effect - C2F6	0.04	tCO_2_e/t	^25^
	Carbon anode use	1.50	tCO_2_/t	^25^
Magnesium	Protection system - refinery	11.52	tCO_2_e/t	^26^
	Protection system - casting	2.68	tCO_2_e/t	^26^
	Electrode use	0.04	tCO_2_/t	^29^
	Solid carbon use	0.53	tCO_2_/t	^29^
Lead	Imperial smelting furnace	0.66	tCO_2_/t	^29^
	Direct smelting	0.25	tCO_2_/t	^29^
	Battery processing	0.20	tCO_2_/t	^29^
Zinc	Imperial smelting furnace	3.12	tCO_2_/t	^29^
	Electro-thermic distillation	0.00	tCO_2_/t	^29^
	Pyrometallurgical process	0.00	tCO_2_/t	^29^

## Data Records

We compile the dataset in an Excel file that consists of 4 sheets. The sheet named “Process-related GHG emissions” which provides industrial process-related GHG emissions for 15 industrial products in China from 1990 to 2020. All the GHG emissions are converted to CO_2_ equivalents by using global warming potential (GWP). The “Activity data” sheet provides the production output of products and the data sources are in the “Activity data source” sheet. The “Emission factors” sheet provides the emission factors we adopted for the emission inventory construction in both CO_2_ equivalent form and original GHG form. The dataset can be accessed on Figshare by the link (10.6084/m9.figshare.20626806.v3)^14^.

**Fig. 2:**
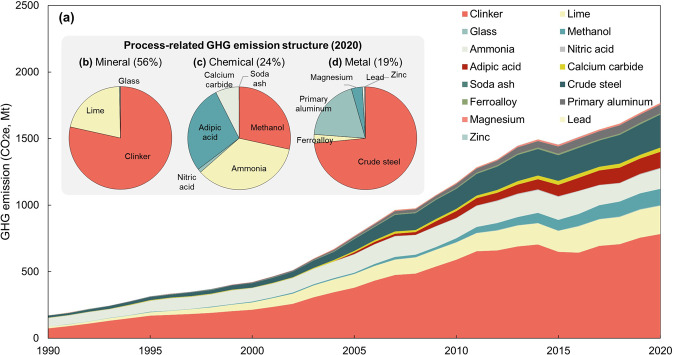
The temporal of industrial process-related GHG emissions in China (1990–2020) and emission structure (2020).

Figure 2a gives an overview of industrial process-related emission in China from 1990 to 2020. The total process-related GHG emissions from China’s industry products can be divided into two stages from 1990 to 2020. The rapid growth period is from 1990 to 2013, in which process-related GHG emissions were driven by the rapid urbanization and industrialization, increasing at 10% per year. During this period, the main industrial product output increased dramatically. For example, clinker production rises from 151 Mt to 1390 Mt and crude steel production rises from 66 Mt to 813 Mt^22,30^. After 2013, the growth rate of GHG emissions slows down, increasing at 3% per year. The total industrial process-related GHG emissions reached 1770 Mt CO_2_e accounting for 17–19% of the national total energy-related emissions in 2020.

The fastest growing sector from 1990–2020 is the metal industry with an annual growth rate of 11%, followed by the mineral industry with an annual growth rate of 8%. Theprocess-related emissions from the metal industry reach 338 Mt CO_2_e and 999 Mt CO_2_e from mineral industry in 2020. The mineral industry provides the primary raw materials for infrastructure and construction. Therefore, process-related emissions grew rapidly up until 2013 due to the rapid growth of infrastructure investment. The emission growth of the chemical industry is slower and more stable with an annual growth rate of 6% from 1990 to 2020 and the total emission was 433 Mt CO_2_e in 2020.

Figure 2b–d show the composition of GHG emissions in 2020 by industrial sectors. The mineral industry contributes the most to industrial process-related emissions, accounting for 56% of China’s total process-related emissions in 2020, in which 44% is from clinker production, 12% is from lime production, and less than 1% is from glass production (Fig. 2b). The chemical industry is responsible for 24% of the national total process-related GHG emissions. The main sources are ammonia (9%), methanol (7%), adipic acid (7%), and calcium carbide (2%) (Fig. 2c). The metal industry contributes 19% of the total industrial process-related emission, and 14% is from using limestone as a solvent to remove impurities from molten iron in the production of crude steel. Aluminum production contributes 4% of total industrial process-related emissions which is mainly generated by using carbon anodes (Fig. 2d).

## Technical Validation

### Uncertainty

The uncertainties in GHG emission inventory may arise from conceptualization,varying models, and input data^15^. Although, due to technical issues, it is difficult to analyze the uncertainties from the perspective of conceptualization and models. We mainly consider the uncertainties from input activity data and emission factors which determine the uncertainty by referring to the estimation given by IPCC guidelines^15^ (Table 3).

**Table 3:** The uncertainty of activity data and emission factors.

Industrial products	Uncertainty of activity data	Uncertainty of emission factor
**Mineral industry**		
Clinker	1.5%	5.5%
Lime	10.0%	6.0%
Glass	5.0%	30.0%
**Chemical industry**		
Methanol	15.0%	30.0%
Ammonia	5.0%	6.0%
Nitric acid	2.0%	5.0%
Adipic acid	2.0%	10.0%
Calcium carbide	5.0%	10.0%
Soda ash	5.0%	0.0%
**Metal industry**		
Limestone use (steelmaking)	10.0%	6.0%
Iron	10.0%	5.0%
Crude steel	10.0%	5.0%
Aluminum	0.5%	10.0%
Magnesium	5.0%	30.0%
Lead	10.0%	50.0%
Zinc	10.0%	50.0%

The majority of activity data is sourced from the statistical yearbooks published by the National Bureau of Statistics, resulting in a relatively low uncertainty of data. However, changes in statistical definition and scope, misreporting or non-reporting, and difficulties in adapting the data collection system to rapidly changing social and economic structures still lead to some degree of uncertainty in these statistics^31–33^. For the products produced by a single route, such as clinker and aluminum, the uncertainty of activity data is lower, ranging from 0.5% to 2%. For the products without officially published output data in China, such as lime, the uncertainty of activity data is 10–15% due to the use of unofficially published estimates^15^. In addition, uncertainty of emission factors is an important source of inventory uncertainty. In most cases, the uncertainty of locally measured emission factors is reduced,  the uncertainty of products with direct country-specific emission factors, such as clinker, lime, and ammonia, etc. is lower, generally less than 10%^15^.

Once the uncertainty of activity data and emission factors for a particular product has been determined, they can be combined to the uncertainty of the GHG emissions inventory for the product, which provides a basis for estimating the uncertainty for the total emission inventory in any given year. IPCC guideline recommends two methods to estimate quantitative uncertainties: error propagation and Monte Carlo simulation^15^. We calculated the uncertainty of the emission inventory by using these two methods.

Two error propagation formulas (Eq.(2) and Eq.(3)) are applied:

2 $${U}_{c}=\sqrt{{U}_{s1}^{2}+{U}_{s2}^{2}+\cdots +{U}_{sn}^{2}}=\sqrt{\mathop{\sum }\limits_{n=1}^{N}{U}_{sn}^{2}}$$

3 $${U}_{c}=\frac{\sqrt{{\left({U}_{s1}\cdot {\mu }_{s1}\right)}^{2}+{\left({U}_{s2}\cdot {\mu }_{s2}\right)}^{2}+\cdots +{\left({U}_{sn}\cdot {\mu }_{sn}\right)}^{2}}}{\left|{\mu }_{s1}+{\mu }_{s2}+\cdots +{\mu }_{sn}\right|}=\frac{\sqrt{{\sum }_{n=1}^{N}{\left({U}_{sn}\cdot {\mu }_{sn}\right)}^{2}}}{\left|{\sum }_{n=1}^{N}{\mu }_{sn}\right|}$$

Where:

*U*_*c*_ represents the combined uncertainty quantities (in percentage);

*U*_*s*1_*…U*_*sn*_ represents the percentage uncertainty of the value *n*.

*μ*_*s*1_*…μ*_*sn*_ represents the quantities of value *n*.

The uncertainty quantities are combined through multiplication in Eq.(2), which is for the combination of the uncertainty from activity data and emission factors of an individual product. The uncertain quantities are combined through addition in Eq.(3), which is for the combination of the uncertainty of all products to form the overall uncertainty of emission inventory. The error propagation process was conducted in Excel 2019.

We also employed Monte Carlo simulations to combine the uncertainties of the activity data and emission factors to estimate the combined uncertainty of the entire emission inventory. We assumed that both activity data and emission factors follow normal distribution with the standard deviations discussed in Table 3. Then, we conducted random sampling on both the activity data and emission factors 100,000 times and obtained 100,000 estimations on the process-related GHG emissions. We used 95% as the confidence interval. The Monte Carlo simulation was conducted in MATLAB R2020b.

**Fig. 3:**
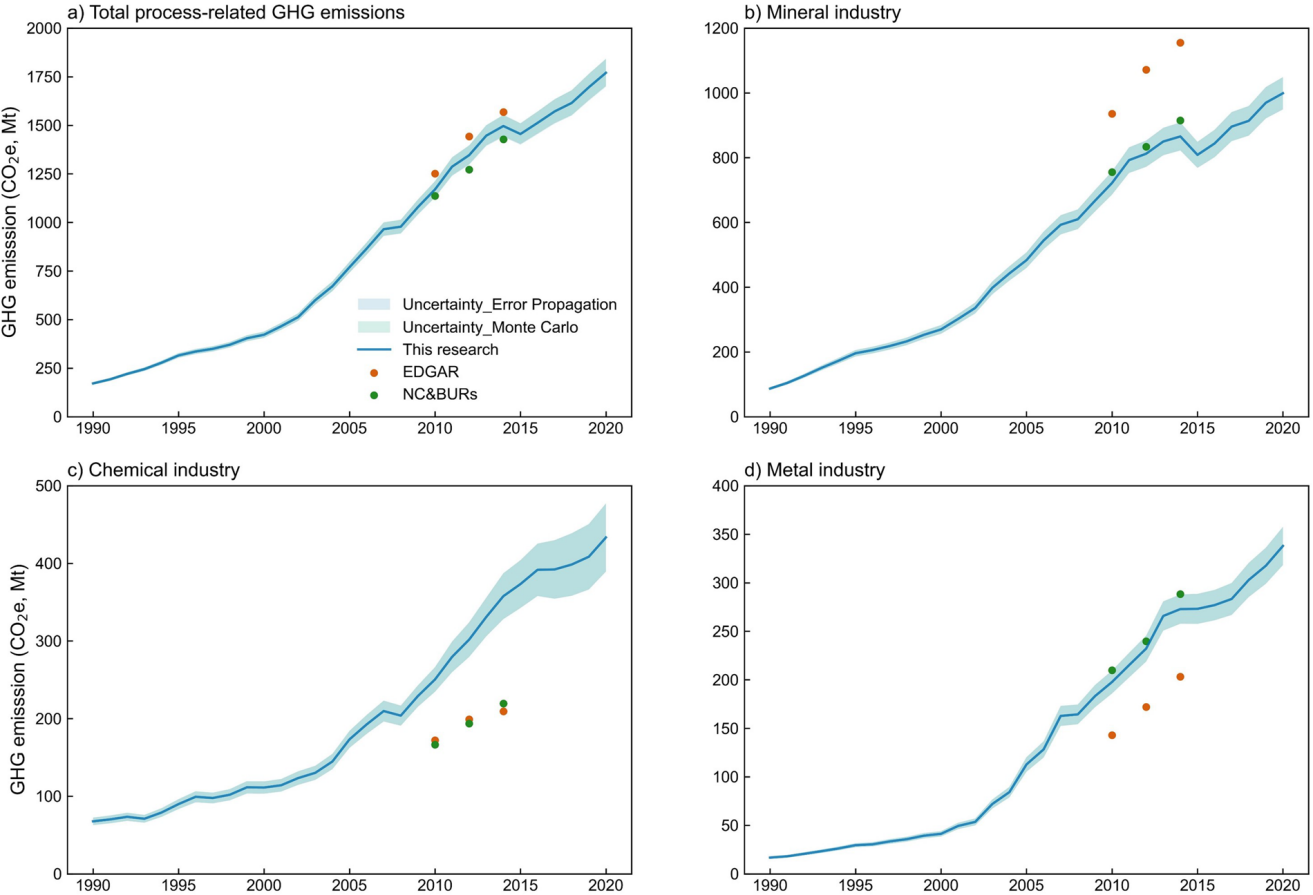
The uncertainty of process-related GHG emissions and comparison of emission inventory for three sectors.

Based on the error propagation formula, the uncertainty of the process-related GHG emission inventory from 1990 to 2020 is 3.6–4.0% (Fig. 3a). The uncertainty obtained from 100,000 Monte Carlo simulations is 3.6–4.1% with a 95% confidence interval (Fig. 3a). IPCC guideline estimates that the uncertainty for less-developed countries is approximately 10%, while for countries with good statistical collection systems, the uncertainty is approximately 5%^15^. This shows that the uncertainties of our process-related GHG emission inventories are much lower than the international average.

### Comparison with existing estimates

Since the uncertainty analysis did not determine any truth values, we further compared the results with other emission inventories. The influential inventories of industrial process-related emissions in China include Emissions Database for Global Atmospheric Research (EDGAR)^34^ and National Communication and Biennial Update Reports of China (NC&BURs)^6,35^. In addition, previous studies also discussed specific industrial products^11,13,28,36^. We compare our results with EDGAR and NC&BURs at the sectoral level since only sectoral level data is publicly available in these two inventories. Furthermore, we also compare our results with studies that focus on the product level.

The EDGAR lists process-related GHG emission inventory of cement, lime, and glass separately in the minerals industry. However, products of the chemical and metal industries are not listed separately. China disclosed its process-related GHG emission in the initial, second and third national communications and the first and second biennial update reports, respectively for 1994, 2005, 2010, 2012 and 2014. The accounting boundaries of emission inventory are consistent for 2010, 2012, 2014 of NC&BURs and EDGAR based on the IPCC guidelines. Therefore, we compared the data in these three years with NC&BURs and EDGAR. The accounting boundaries of EDGAR, NC&BURs and this research are shown in Table 4 and the emission comparison is demostrated in Fig. 3.

**Table 4:** Accounting boundary comparison.

		NC&BURs					EDGAR	This research
		1994	2005	2010	2012	2014		
Mineral Industry	Cement	✓	✓	✓	✓	✓	✓	✓
	Lime	✓	✓	✓	✓	✓	✓	✓
	Glass			✓	✓	✓	✓	✓
Chemical Industry	Ammonia			✓	✓	✓	✓	✓
	Calcium carbide	✓	✓	✓	✓	✓		✓
	Soda ash			✓	✓	✓		✓
	Nitric acid		✓	✓	✓	✓		✓
	Adipic acid		✓	✓	✓	✓		✓
	Methanol							✓
Metal Industry	Steel	✓	✓	✓	✓	✓	✓	✓
	Ferroalloy			✓	✓	✓	✓	✓
	Aluminum		✓	✓	✓	✓		✓
	Magnesium		✓	✓	✓	✓		✓
	Lead			✓	✓	✓		✓
	Zinc			✓	✓	✓		✓

For the mineral industry, our result is close to NC&BURs but 23% lower than EDGAR (Fig. 3b). There are two explanations for the overestimation of EDGAR: First, different clinker to cement ratios (CCR) were used. CCR in China is significantly lower than the global average level^9^. Using the default value of 65% recommended by IPCC guideline^15^ leads to overestimation. In addition, the estimation errors may also be induced by using proxies for downscalling of large-scale emission data^34^. The unrepresentative proxies may lead to bias in the results.

For the chemical industry, our results are significantly higher than those of EDGAR and NC&BURs (Fig. 3c). The main reason for such large variances is the difference in accounting scope. As shown in Table 4, our research includes more products in the chemical industry than in EDGAR and NC&BURs, especially for methanol, which is not accounted in either inventory. The emission factors selected also contribute to the variation. The emission factors provided by IPCC guidelines were adopted by both EDGAR and NC&BURs for the chemical industry. However, these emission factors are based on natural gas as the default feedstock, which is the common practice in Europe. In contrast, many Chinese chemical products are based on coal. For example, according to the expert consultation in our study, 80% of methanol prodution uses coal-based process. Therefore, the emission factors of chemical products like methanol and ammonia in China is almost twice as high as those in Europe.

For the metal industry, differences in accounting scope contribute to the variation. Since EDGAR does not include process-related emissions from nonferrous metals such as aluminum, magnesium, lead, and zinc, its results are about 26–30% lower than those of NC&BURs and this study. However, the difference between our accounting results and NC&BURs is less than 5% (Fig. 3d).

For an individual product, we mainly compare the emission factors used in this study with others:

There is a relatively broad range of studies on clinker, and the emission factor of clinker in China ranges from 0.49–0.53 tCO_2_/t clinker^9,10,26,27,36,37^. The difference among these studies is within 8%. The emission factor used in our research is 0.50 tCO_2_/t clinker, which falls in the middle of the above range.

Shan *et al*. discussed process-related CO_2_ emissions from lime industry in China^11^. The emission factor they adopted is 0.683 tCO_2_/t lime, which is consistent with this research since both are derived from the recommended value by the NDRC. According to the IPCC guideline, the default emission factor for lime is 0.75 tCO_2_/t lime in the absence of country-specific data, which is 9.8% higher than the default emission factor recommended by NDRC.

The default emission factor for glass from IPCC guideline (0.2 tCO_2_/t glass) was used in the emission inventory of Cui’s study^13^. This value is derived based on a typical raw material mix, in which limestone and soda ash. The main components that produce CO_2_ emissions in glass production, account for 8.6% and 20.0% in total feedstock^15^. However, according to Hu’s investigation of glass factories throughout China, the share of limestone and soda ash may only account for 8.4% and 8.5% respectively in China’s glass production feedstock^28^. Therefore, Hu *et al*. provide the emission factor of 0.07 tCO_2_/t glass. Although this value is lower than the IPCC default value, it is more applicable to China.

As previously discussed, coal-based chemical products have a higher process-related emission factor (in this research, 2.97 tCO_2_/t ammonia) compared to the default value from IPCC guideline, which is (1.70 tCO_2_/t ammonia) based on natural gas production.

Cui *et al*. used the emission factor given by the IPCC guideline (0.14 tCO_2_/t soda ash) for the emission inventory of soda ash^13^. However, this emission factor for soda ash is produced from natural alkali mines. According to expert consultation from the China Soda Industry Association (CSIA), only about 5% of soda ash production in China is currently from natural alkali mines. Therefore, the integrated emission factor for soda ash used in our paper is 0.007 tCO_2_/t soda ash.

## Data Availability

All the emission accounting is performed in Excel.
